# HMMBinder: DNA-Binding Protein Prediction Using HMM Profile Based Features

**DOI:** 10.1155/2017/4590609

**Published:** 2017-11-14

**Authors:** Rianon Zaman, Shahana Yasmin Chowdhury, Mahmood A. Rashid, Alok Sharma, Abdollah Dehzangi, Swakkhar Shatabda

**Affiliations:** ^1^Department of Computer Science and Engineering, United International University, Dhaka, Bangladesh; ^2^School of Computing, Information and Mathematical Sciences, The University of the South Pacific, Suva, Fiji; ^3^Institute for Integrated and Intelligent Systems, Griffith University, Brisbane, QLD, Australia; ^4^School of Engineering and Physics, The University of the South Pacific, Suva, Fiji; ^5^RIKEN Center for Integrative Medical Sciences, Yokohama, Japan; ^6^Department of Computer Science, Morgan State University, Baltimore, MD, USA

## Abstract

DNA-binding proteins often play important role in various processes within the cell. Over the last decade, a wide range of classification algorithms and feature extraction techniques have been used to solve this problem. In this paper, we propose a novel DNA-binding protein prediction method called HMMBinder. HMMBinder uses monogram and bigram features extracted from the HMM profiles of the protein sequences. To the best of our knowledge, this is the first application of HMM profile based features for the DNA-binding protein prediction problem. We applied Support Vector Machines (SVM) as a classification technique in HMMBinder. Our method was tested on standard benchmark datasets. We experimentally show that our method outperforms the state-of-the-art methods found in the literature.

## 1. Introduction

DNA-binding proteins play a vital role in various cellular processes. They are essential in transcriptional regulation, recombination, genome rearrangements, replication, repair, and DNA modification [1]. Proteins which make bond with DNA in both eukaryotes and prokaryotes while performing like activators or repressors are DNA-binding proteins. It has been observed that the percentages of prokaryotes and eukaryotes protein that can bind to DNA are only 2-3% and 4-5%, respectively [2, 3]. There have been a wide variety of experimental methods such as* in vitro* methods [4, 5] like filter binding assays, chromatin immunoprecipitation on microarrays (ChIP-chip) genetic analysis, and X-ray crystallography which are used to predict DNA-binding proteins. However, these methods are proven to be expensive and time consuming. Therefore, there is a growing demand to find a fast and cost effective computational method to solve this problem.

Most of the computational methods used in the literature to predict DNA-binding proteins formulated the problem as a supervised learning problem. Practically, the number of known DNA-binding proteins is very small compared to the large non-DNA-binding proteins and unknown proteins. DNA-binding protein prediction is often modeled as a binary class classification problem where given a protein sequence as input the task is to predict whether the protein is DNA-binding or not. Note that the challenge here is to select a proper dataset for training and testing incorporating the imbalanced situation. Many supervised learning algorithms have been used in the literature to solve the problem. Among them, Artificial Neural Networks (ANN) [6], Support Vector Machines (SVM) [7, 8], ensemble methods [9], Nave Bayes classifier [10], Random Forest [11], Convolutional Neural Networks [12], Logistic Regression [13], AdaBoost Classifier [5], and so on are well-regarded. Support Vector Machines (SVM) are one of the best performing classifiers used for DNA-binding protein identification [7, 8, 14, 15].

A great number of web based tools and methods are developed for DNA-binding protein prediction and are available for use. In this paper, we would like to mention several of them: DNABinder [7], DNA-Prot [16], iDNA-Prot [11], iDNA-Prot|dis [14], DBPPred [17], iDNAPro-PseAAC [8], PseDNA-Pro [18], Kmer1 + ACC [19], Local-DPP [20], SVM-PSSM-DT [21], PNImodeler [22], CNNsite [12], and BindUP [23]. Most of these methods have used sequence, profile, or structure based features. In structural feature based methods in the literature, features used were structural motifs, electrostatic potential, the dipole moment, and *α*-carbon only models [13, 24, 25]. On the other hand, sequence based methods often depended on the PSSM profile based information or pseudo-amino-acid compositions [8, 14, 15, 17, 20, 26, 27]. In [28], HMM based profiles were used for generating features for protein fold recognition.

In this paper, we propose HMMBinder, a novel DNA-binding protein prediction tool using HMM profile based features of a protein sequence. Our method uses monogram and bigram features derived from the HMM profile which shows effectiveness compared to the PSSM or sequence based features. We also use SVM as the classifier and standard benchmark datasets to test our method. Using the standard evaluation metrics, our method significantly improves over the state-of-the-art methods and the features used in the literature. We also developed a web server that is publicly available at http://brl.uiu.ac.bd/HMMBinder.

The rest of the paper is organized following the general 5-step guideline suggested in [29] for protein attribute prediction. First, benchmark datasets selected for this problem are described followed by a description of the protein representation by extraction of features. Then we describe the classification algorithm that we selected for our approach followed by the performance evaluation techniques deployed in this paper. Lastly, we describe the web server that we developed for this problem. The results section presents the details of the experimental results followed by an analytical discussion. The paper concludes with a summary and indication of future work.

## 2. Methods and Materials

**Figure 1:**
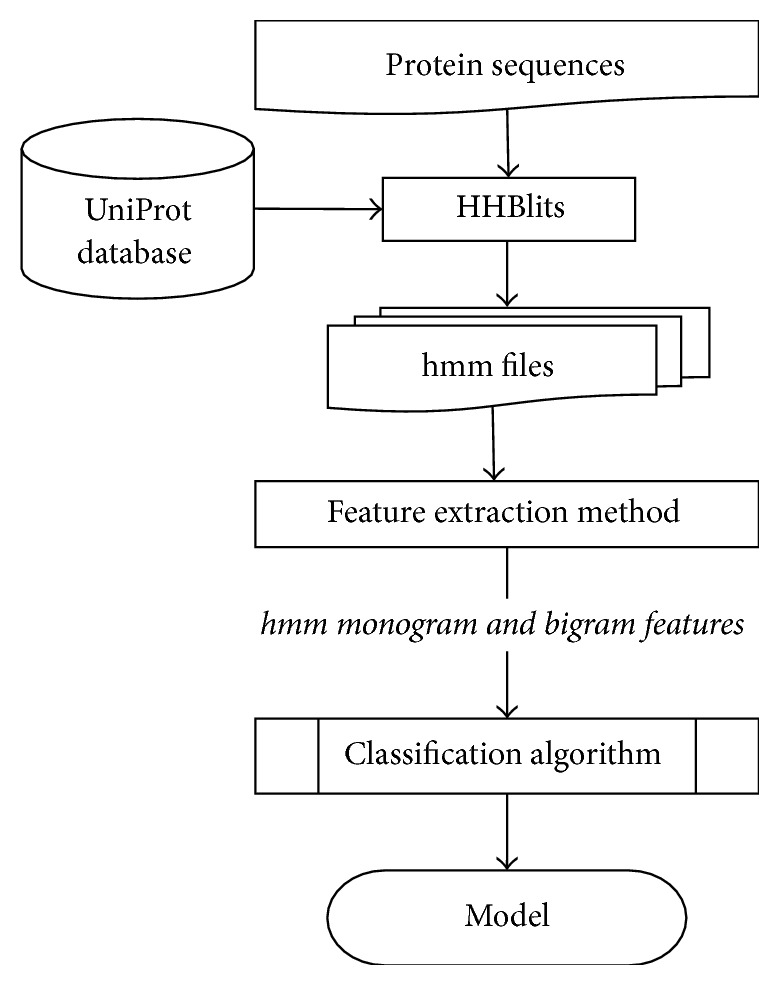
System diagram of HMMBinder.

In this section, we provide the details of the materials and methods of this paper.  Figure 1 provides a system diagram of our proposed method. For the training phase, all the protein sequences are fed to HHBlits [30], a sequence-to-sequence alignment software using the latest UniProt database. HHBlits produces HMM file as output which is then used by our feature extraction method to generate monogram and bigram features. Monogram and bigram features are concatenated together and then used as training feature set to train the classifier. We use SVM with linear kernel as the classification algorithm and a trained model is stored for the testing phase. Testing phase is also similar to the training phase; however, the labels for the test dataset are not given to the classifier. This stored model is also used for the web server implementation of HMMBinder.

### 2.1. Datasets

Selection of benchmark datasets is essential in classification and prediction design. In this paper we use a popular benchmark dataset called* benchmark1075* to train our model. Later we test the performance using cross validation and on a separate independent test set known as* independent186* dataset. This section provides a brief overview of these two datasets. Both of these datasets are widely used in the literature of DNA-binding protein prediction literature [8, 14, 18, 20, 31].

#### 2.1.1. Dataset Benchmark1075

This dataset was first introduced in [14]. This dataset consists of 1075 protein sequences. Among them, 525 are DNA-binding and 550 are non-DNA-binding protein sequences. All the protein sequences were taken from PDB [32]. This dataset is one of the largest DNA-binding protein prediction datasets and thus suitable for training purpose.

#### 2.1.2. Dataset Independent186

Lou et al. [17] constructed this independent dataset consisting of 93 DNA-binding and 93 non-DNA-binding protein sequences. They used BLASTCLUST [33] on the benchmark dataset to remove the sequences that have more that 25% of similarity.

### 2.2. Feature Extraction

The training dataset *𝕊* used for a binary classification problem consists of two types of instances: positive and negative. Formally,

(1) $$\begin{array}{c} S=S^{+}\cup S^{-}. \end{array}$$

Next, the task is to represent each protein instance as feature vectors suitable for training. The idea is to represent each of the protein instances as a vector of features.

(2) $$\begin{array}{c} P=\left[\;f_{1},f_{2},\ldots ,f_{n}\;\right]. \end{array}$$

Here, a protein, *P* ∈ *𝕊*, is shown as a feature vector with dimension *n*. Most of the methods in the literature of DNA-binding protein prediction use either sequence and PSSM profile based features or structure based features. To the best of our knowledge, there has been no application of features using HMM profiles. In this paper, we have used HHBlits [30] to generate HMM profiles. HMM profiles are comparatively more effective [30, 34] for remote homology detection. HMM profiles were generated using four iterations of HHBlits with a cutoff value set to 0.001 using the latest UniProt database [35]. HMM profiles are *L* × 20 matrix produced by HHBlits. These 20 values are the substitution probability of each type of amino-acid residue along the protein sequence at each position. These values are first converted to linear probabilities using the following formula:

(3) $$\begin{array}{c} p=2^{-N/1000}. \end{array}$$

We generated two types of features, monogram and bigram, using the generated HMM profile matrix noted here as *H*. We provide a brief description of monogram and bigram features extracted from the HMM profile matrix.

#### 2.2.1. Monogram Features

Monogram features [36] are calculated taking the normalized sum of the column wise substitution probability values. Size of these feature group is 20 because of 20 different amino acids. The feature can be defined formally as follows:

(4) $$\begin{array}{c} HMM\text{-}Monogram\left(\;j\;\right)=\frac{1}{L}\overset{L}{\underset{i=1}{\sum }}H_{i,j}. \end{array}$$

Note that values of *j* are dependent on the columns; that is, 1 ≤ *j* ≤ 20. Here, *H*_*ij*_ are the values in the *i*th row and *j*th column of the matrix. We denote monogram features as *M* which is a vector of the form *M* = [*m*(1), *m*(2),…, *m*(20)].

#### 2.2.2. Bigram Features

Bigram features have been successfully used in the literature for protein attribute prediction [37]. Bigram features are normalized bigrams taken for all pairs of columns. Hence the total number of features generated from this group is 400. Bigram features are generated using the following formula:

(5) $$\begin{array}{c} HMM\text{-}Bigram\left(\;j,k\;\right)=\frac{1}{L-1}\overset{L}{\underset{i=1}{\sum }}H_{i,j}H_{i+1,k}. \end{array}$$

Here *j* and *k* denote the column pairs for which the bigram is calculated and are in the ranges 1 ≤ *j* ≤ 20 and 1 ≤ *k* ≤ 20. We denote this feature vector as *B*, where *B* has the form of *B* = [*b*(1,1), *b*(1,2),…, *b*(1,20), *b*(2,1),…, *b*(20,20)].

We also generate Positive Specific Scoring Matrix (PSSM) profiles for each of the protein sequences using PSI-BLAST [38]. PSSMs were generated using three iterations of PSI-BLAST using the nr database with a cutoff value of 0.001. PSSM profiles also have a similar form to HMM profiles which is a matrix of the same dimension and each of the matrix values denotes substitution probabilities. We generate monogram and bigram features from PSSM files as well. These PSSM based monogram and bigram features are well used in the literature [36, 37, 39–42]. Note that all the monogram features are vectors of size 20 and bigram features are vectors of size 400. We have also used a combination of the monogram and bigram features which is a vector of size 420.

### 2.3. Support Vector Machine

We have used Support Vector Machines (SVM) as our classification technique. SVM is successfully used in protein attribute prediction in general [28, 39, 43] and particularly in DNA-binding protein prediction [7, 8]. SVM is maximum margin classifier that attempts to learn a hyperplane from the training samples that separates the positive and negative data points in a binary classification problem. The hyperplane that is selected is the one for which the separation width or the margin is maximum and the nature of the hyperplane depends on the kernel functions used. SVM generally tries to optimize a multiplier function that goes as follows:

(6) $$\begin{array}{c} L=\underset{\alpha }{\arg max}\underset{j}{\sum }\alpha_{j}-\frac{1}{2}\underset{j,k}{\sum }\alpha_{j}\alpha_{k}y_{j}y_{k}\phi \left(\;{\vec{x}}_{j}\cdot {\vec{x}}_{k}\;\right). \end{array}$$

The prediction of a SVM classifier is defined as follows:

(7) $$\begin{array}{c} h\left(\;\vec{x}\;\right)=\mathrm{sign}\left(\;\underset{j}{\sum }\alpha_{j}y_{j}\left(\;\vec{x}\cdot {\vec{x}}_{j}\;\right)-b\;\right). \end{array}$$

Here the transformation of the data points by the function *ϕ* could be linear, polynomial, or any other kernel functions. In this paper, we explored linear and radial basis function (RBF) kernels. Linear kernel is of the following form:

(8) $$\begin{array}{c} K\left(\;{\vec{x}}_{i},{\vec{x}}_{j}\;\right)={\left(\;{\vec{x}}_{i}\cdot {\vec{x}}_{j}+c\;\right)}^{d}. \end{array}$$

Here *d* = 1 for the linear kernels. RBF kernels follow the following definition:

(9) $$\begin{array}{c} K\left(\;{\vec{x}}_{i},{\vec{x}}_{j}\;\right)=e^{\left(-\right)\left({\left\|{\vec{x}}_{i}-{\vec{x}}_{j}\right\|}^{2}/2\sigma^{2}\right)}. \end{array}$$

Often slack variables are used along with the maximum margin SVM classifier to allow generalization error depending on a parameter *C*.

### 2.4. Performance Evaluation

A good number of effective evaluation metrics have been suggested for use in single valued and multivalued classification and prediction [29, 44]. In the literature of DNA-binding protein prediction, we have found that the most widely used metrics are accuracy, sensitivity, specificity, MCC, auROC, and auPR values. In this section, we first provide a description of these evaluation metrics used in this paper.

(10) $$\begin{array}{c} Accuracy=\frac{TP+TN}{TP+TN+FP+FN}. \end{array}$$

This first measure, accuracy, is the ratio or percentage of correctly classified negative or positive instances from a given number of protein instances. Here TP is the total number of true positives or correctly classified positive samples and TN is the correctly classified negative samples. FP and FN are incorrectly classified positive and negative instances, respectively. Sensitivity is the true positive rate or the ratio of true positives to the total number of positive examples. Sensitivity is defined in the following equation:

(11) $$\begin{array}{c} Sensitivity=\frac{TP}{TP+FN}. \end{array}$$

Specificity on the other hand is the true negative rate and can be defined as the following equation:

(12) $$\begin{array}{c} Specificity=\frac{TN}{TN+FP}. \end{array}$$

All these three measures have a maximum value of 1 which is the best classifier and a minimum value of 0 meaning the worst classifier. Mathew's Correlation Coefficient (MCC) denotes how good a binary classification is working. The value of MCC is in the range [−1, +1]. A perfect classifier should have a maximum MCC value of +1. MCC is defined as the following equation:

(13) MCC=TP×TN−FP×FNTP+FPTP+FNTN+FPTN+FN.

Note that all these metrics for probabilistic outputs depend on the threshold set for the classifiers. Two other metrics not dependent on thresholds are area under receiver operating characteristic curve (auROC) and area under precision-recall curve (auPR). The value of auROC and auPR has maximum value of 1 for the perfect classifier. ROC curve plots true positive rate against false positive rate at different threshold values and precision-recall curve plots precision against recall.

To reduce the training bias, several sampling methods are proposed in the literature [45] and widely used for protein attribute prediction [29]. In this paper, we have used 10-fold cross validation and jack-knife tests which are widely used in the literature of DNA-binding protein prediction [8, 11, 14, 17].

## 3. Results and Discussion

In this section, we present the results of the experiments that were carried out in this study. All the methods were implemented in Python3.4 programming language. The Scikit-learn library [46] of python was used for implementing the machine learning algorithms. All experiments were conducted on computing services provided by CITS, United International University.

### 3.1. Effect of HMM Based Features

**Figure 2:**
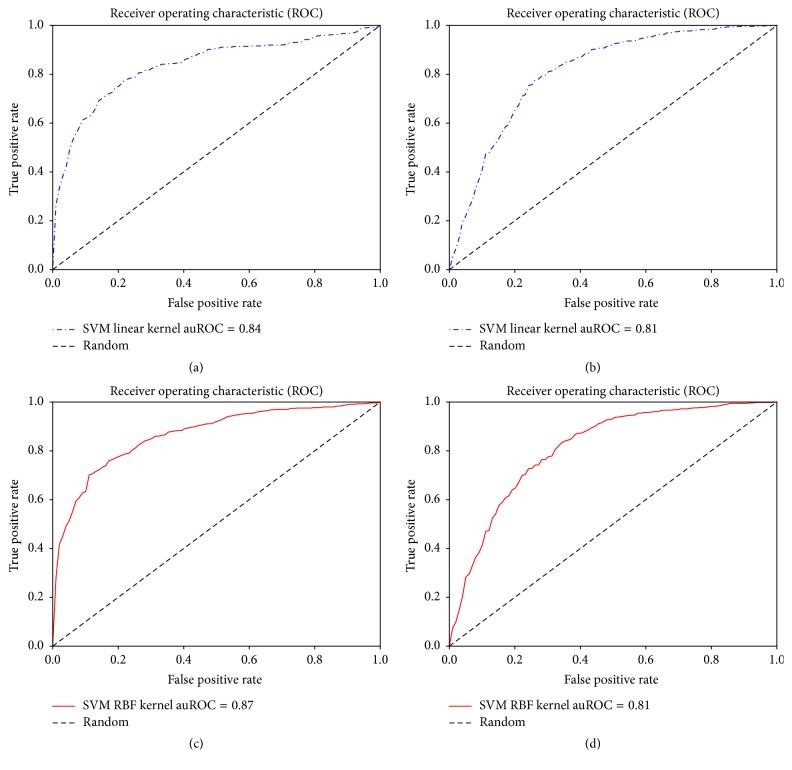
Using monogram features. Receiver operating characteristic curves for (a) SVM linear kernel classifier using HMM-Monogram features, (b) SVM linear kernel classifier using PSSM-Monogram features, (c) SVM RBF kernel classifier using HMM-Monogram features, and (d) SVM RBF kernel classifier using PSSM-Monogram features.

**Figure 3:**
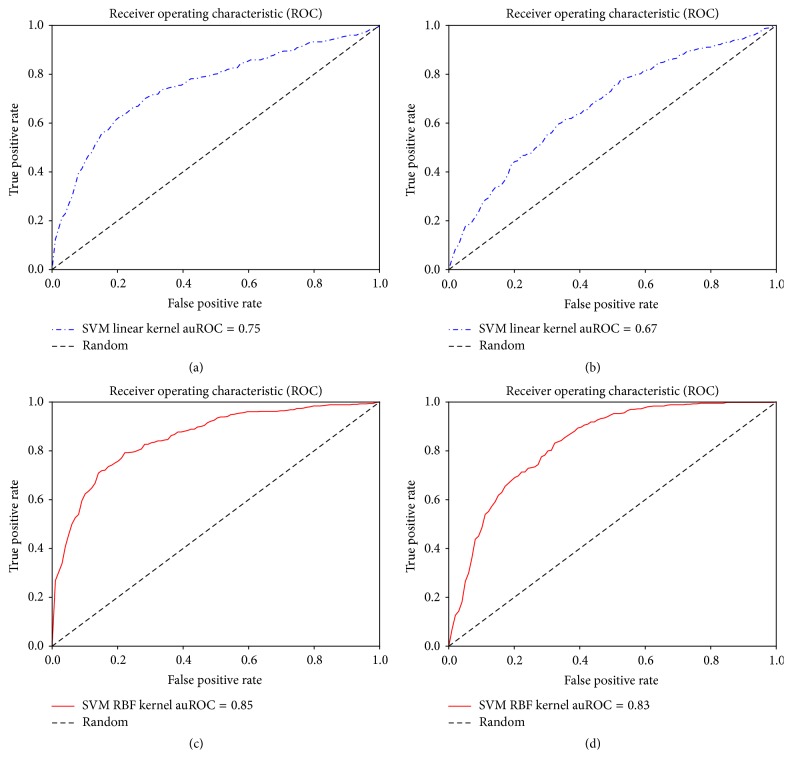
Using bigram features. Receiver operating characteristic curves for (a) SVM linear kernel classifier using HMM-Bigram features, (b) SVM linear kernel classifier using PSSM-Bigram features, (c) SVM RBF kernel classifier using HMM-Bigram features, and (d) SVM RBF kernel classifier using PSSM-Bigram features.

**Figure 4:**
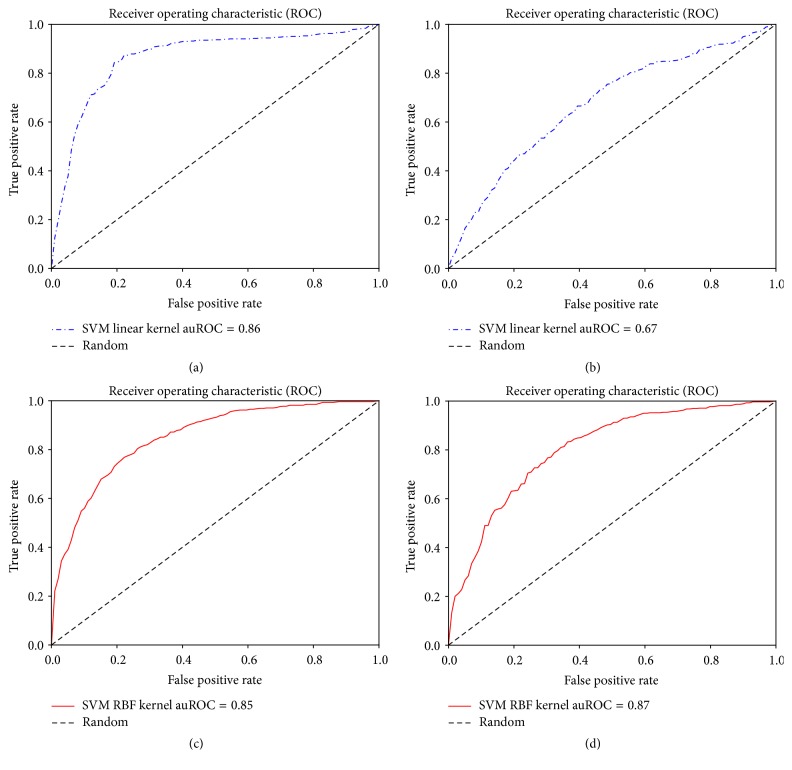
Using (Mono + Bi)gram features. Receiver operating characteristic curves for (a) SVM linear kernel classifier using HMM-Mono + Bigram features, (b) SVM linear kernel classifier using PSSM-Mono + Bigram features, (c) SVM RBF kernel classifier using HMM-Mono + Bigram features, and (d) SVM RBF kernel classifier using PSSM-Mono + Bigram features.

We have run a number of experiments to test the effectiveness of the HMM profile based features on the benchmark dataset. We have six groups of features extracted for this experiment: PSSM-Monogram, PSSM-Bigram, PSSM-Mono + Bigram, HMM-Monogram, HMM-Bigram, and HMM-Mono + Bigram. Each of these feature sets is tested with SVM classifiers using linear and RBF kernels. We further tested the performances of these features using two ensemble classifiers: Random Forest and AdaBoost Classifiers. For these experiments we have performed 10-fold cross validation. The results in terms of accuracy, sensitivity, specificity, auPR, auROC, and MCC are reported. Only the average of these values is reported in Table 1. Note that the best results were found using the combination of HMM-Monogram and Bigram features and 82.87% accuracy was achieved using SVM linear kernels. In each case of the SVM linear kernel, HMM based features achieved better accuracy compared to PSSM based features. Similar results could be noticed for auROC, MCC, and sensitivity analysis. Specificity, auROC, and auPR are slightly improved in the experiments with SVM with RBF kernels. We also show the ROC curves for each of these experiments in Figures 2, 3, and 4.

### 3.2. Comparison with Other Methods

We have compared the performance of HMMBinder with several previous methods and tools used for DNA-binding protein prediction on the benchmark dataset* benchmark1075*. They are DNABinder [7], DNA-Prot [16], iDNA-Prot [11], iDNA-Prot|dis [14], DBPPred [17], iDNAPro-PseAAC [8], PseDNA-Pro [18], Kmer1 + ACC [19], and Local-DPP [20]. The results reported in this paper for these methods are taken from [8, 20]. The comparisons were made in terms of accuracy, sensitivity, specificity, MCC, and auROC. To make a fair comparison with the other methods, we performed jack-knife test as done in earlier studies and the results are reported in Table 2.

The best values in Table 2 are shown in bold faced fonts. The results show a clear margin of more than 7% improvement of accuracy over the previous best method, Local-DPP [20]. Similar improvements were found in other metrics too. Particularly, MCC is increased by 22% compared to the previous best method.

We further experimented to test the effectiveness of HMMBinder on the independent test set also. These results are shown in Table 3. Here the results are not the best but among the best. In terms of accuracy, our results are almost similar to iDNAPro-PseAAC [8]. Their results were significant in the benchmark dataset and were similar to ours in the independent dataset. Specificity value of HMMBinder was among the best and only second to DNA-Threader which failed miserably in terms of accuracy. Considering the difficulty level of the independent dataset, we believe that our method has not been overtrained on the benchmark dataset and the performance is promising and can be claimed as a generalized method after training and testing. Based on these results, we decided to build the web application based on the model trained on the benchmark dataset.

Note that the results on the independent dataset are comparative but not improved in comparison to the state-of-the-art methods. The main focus of this research was to build a classifier based on HMM profiles instead of the PSSM profile based features and we experimentally showed the effectiveness of the HMM profile based features over PSSM. In the future, we aim to focus on the independent dataset to perform better.

Additionally, we would like to highlight two points. Firstly, the datasets that we used were filtered using BLASTCLUST. It is important to remove the sequences with similarity more than 25% from the dataset before applying the training and testing methods. We used the dataset proposed by Lou et al. [17], a widely accepted standard independent test dataset where the sequences with similarity of 25% or more with other sequences had been removed. We believe it would be interesting to see the effects of the other heuristic, CLUSTALW [47]. Secondly, feature selection methods are gaining much popularity in case of bioinformatics data and supervised machine learning. We believe that using sophisticated feature selection methods, such as maximum relevance minimum redundancy (mRMR) [48] and maximum relevance maximum distance (MRMD) [49], could improve the results further.

### 3.3. Web Server Implementation

We have implemented a web based application based on the proposed method. We call this HMMBinder. This is readily available to use at http://brl.uiu.ac.bd/HMMBinder. The server was implemented using PHP web programming language in the front end and python based prediction engine at the backend. The software requires an HMM profile as input to the tools that can be generated by HHBlits. The features are extracted automatically by the python program and the predicted value from a trained model is shown in the web form. The web site contains a “read me” guide and the necessary information required to run the application.

## 4. Conclusion

In this paper, we have introduced HMMBinder, a HMM profile based method for the DNA-binding protein prediction problem. We have used monogram and bigram features extracted from the HMM profiles generated by HHBlits and a SVM classification algorithm to train our data on a standard benchmark dataset. Our method is able to make considerable improvement over the other state-of-the-art methods on this dataset and performed comparably well in the independent dataset. We have also established a web based application for our method that is trained on the benchmark dataset. In the future, we wish to extract more effective features and generate larger dataset to train our model to be able to improve the results on the independent dataset. We believe there is a scope of improvement.

## Figures and Tables

**Table 1:** Comparison of performances of different features and SVM kernels on the benchmark dataset using 10-fold cross validation.

Features	Accuracy	Sensitivity	Specificity	auPR	MCC	auROC
*SVM with linear kernel*						

HMM-Monogram	76.77%	**0.8420**	0.6976	0.6931	0.5367	0.8358
PSSM-Monogram	74.74%	0.6636	0.8362	0.8368	0.5040	0.8105

HMM-Bigram	70.59%	0.7071	0.7049	0.7060	0.4095	0.7511
PSSM-Bigram	62.20%	0.6454	0.5973	0.6025	0.2502	0.6703

HMM (Mono + Bi)	**82.87%**	0.8150	**0.8415**	**0.8428**	**0.6538**	**0.8639**
PSSM (Mono + Bi)	72.40%	0.7364	0.7120	0.7136	0.4486	0.8028

*SVM with RBF kernel*						

HMM-Monogram	**78.83%**	**0.8227**	0.7559	0.7535	**0.5761**	**0.8667**
PSSM-Monogram	73.71%	0.6890	0.7880	0.7903	0.4771	0.8121

HMM-Bigram	76.68%	0.7052	**0.8251**	0.8253	0.5283	0.8318
PSSM-Bigram	74.92%	0.7490	0.7495	0.7516	0.4966	0.8166

HMM (Mono + Bi)	77.43%	0.7129	0.8324	**0.8329**	0.5440	0.8496
PSSM (Mono + Bi)	72.40%	0.7363	0.7120	0.7136	0.4486	0.8028

*Random Forest*						

HMM-Monogram	**74.44%**	**0.7938**	**0.6976**	**0.6936**	**0.4871**	0.8243
PSSM-Monogram	66.14%	0.7290	0.5895	0.5862	0.3173	0.7332

HMM-Bigram	72.19%	0.7553	0.6903	0.6880	0.4400	**0.8273**
PSSM-Bigram	71.00%	0.7854	0.6300	0.6305	0.4174	0.7833

HMM (Mono + Bi)	74.43%	**0.7938**	**0.6976**	0.6931	**0.4871**	0.8218
PSSM (Mono + Bi)	72.68%	0.7909	0.6589	0.6645	0.4557	0.7698

*AdaBoost*						

HMM-Monogram	73.31%	0.7013	0.7632	0.7603	0.4579	0.8026
PSSM-Monogram	67.07%	0.7654	0.5703	0.5737	0.3448	0.7157

HMM-Bigram	73.97%	0.7360	0.7432	0.7396	0.4762	0.8063
PSSM-Bigram	70.53%	0.7436	0.6647	0.6708	0.4116	0.7710

HMM (Mono + Bi)	**78.00%**	**0.7803**	**0.7795**	**0.7732**	**0.5532**	**0.8577**
PSSM (Mono + Bi)	70.07%	0.7327	0.6666	0.6687	0.4005	0.7887

**Table 2:** Comparison of performance of the proposed method with other state-of-the-art predictors using jack-knife test on the benchmark dataset.

Method	Accuracy	Sensitivity	Specificity	MCC	auROC
iDNAPro-PseAAC	76.76%	0.7562	0.7745	0.53	0.8392
DNABinder (dimension 21)	73.95%	0.6857	0.7909	0.48	0.8140
DNABinder (dimension 400)	73.58%	0.6647	0.8036	0.47	0.8150
DNA-Prot	72.55%	0.8267	0.5976	0.44	0.7890
iDNA-Prot	75.40%	0.8381	0.6473	0.50	0.7610
iDNA-Prot|dis	77.30%	0.7940	0.7527	0.54	0.8310
PseDNA-Pro	76.55%	0.7961	0.7363	0.53	—
Kmer1 + ACC	75.23%	0.7676	0.7376	0.50	0.8280
Local-DPP	79.20%	0.8400	0.7450	0.59	—
HMMBinder	**86.33%**	**0.8707**	**0.8555**	**0.72**	**0.9026**

**Table 3:** Comparison of performance of the proposed method with other state-of-the-art predictors on the independent dataset.

Method	Accuracy	Sensitivity	Specificity	MCC	auROC
iDNAPro-PseAAC	69.89%	0.7741	0.6237	0.402	0.7754
iDNA-Prot	67.20%	0.6770	0.6670	0.344	—
DNA-Prot	61.80%	0.6990	0.5380	0.240	—
DNABinder	60.80%	0.5700	0.6450	0.216	0.6070
DNABIND	67.70%	0.6670	0.6880	0.355	0.6940
DNA-Threader	59.70%	0.2370	**0.9570**	0.279	—
DBPPred	76.90%	0.7960	0.7420	0.538	0.7910
iDNA-Prot|dis	72.00%	0.7950	0.6450	0.445	**0.7860**
Kmer1 + ACC	70.96%	0.8279	0.5913	0.431	0.7520
Local-DPP	**79.00%**	**0.9250**	0.6560	**0.625**	—
HMMBinder	69.02%	0.6153	0.7634	0.394	0.6324

## References

[1] Berman H. M., Thornton J. M., Luscombe N. M., Austin S. E. (2000). An overview of the structures of protein-dna complexes. *Genome Biology*.

[2] Stawiski E. W., Gregoret L. M., Mandel-Gutfreund Y. (2003). Annotating nucleic acid-binding function based on protein structure. *Journal of Molecular Biology*.

[3] Jones S., Thornton J. M., Shanahan H. P., Garcia M. A. (2004). Identifying DNA-binding proteins using structural motifs and the electrostatic potential. *Nucleic Acids Research*.

[4] Jaiswal R., Singh S. K., Bastia D., Escalante C. R. (2015). Crystallization and preliminary X-ray characterization of the eukaryotic replication terminator Reb1-Ter DNA complex. *Acta Crystallographica Section F:Structural Biology Communications*.

[5] Langlois R. E., Lu H. (2010). Boosting the prediction and understanding of DNA-binding domains from sequence. *Nucleic Acids Research*.

[6] Ahmad S., Gromiha M. M., Sarai A. (2004). Analysis and prediction of DNA-binding proteins and their binding residues based on composition, sequence and structural information. *Bioinformatics*.

[7] Kumar M., Gromiha M. M., Raghava G. P. S. (2007). Identification of DNA-binding proteins using support vector machines and evolutionary profiles. *BMC Bioinformatics*.

[8] Liu B., Wang S., Wang X. (2015). DNA binding protein identification by combining pseudo amino acid composition and profile-based protein representation. *Scientific Reports*.

[9] Song L., Li D., Zeng X., Wu Y., Guo L., Zou Q. (2014). nDNA-prot: identification of DNA-binding proteins based on unbalanced classification. *BMC Bioinformatics*.

[10] Yan C., Terribilini M., Wu F., Jernigan R. L., Dobbs D., Honavar V. (2006). Predicting DNA-binding sites of proteins from amino acid sequence. *BMC Bioinformatics*.

[11] Lin W.-Z., Fang J.-A., Xiao X., Chou K.-C. (2011). iDNA-prot: identification of DNA binding proteins using random forest with grey model. *PLoS ONE*.

[12] Zhou J., Lu Q., Xu R., Gui L., Wang H. CNNsite: Prediction of DNA-binding residues in proteins using Convolutional Neural Network with sequence features.

[13] Szilágyi A., Skolnick J. (2006). Efficient Prediction of Nucleic Acid Binding Function from Low-resolution Protein Structures. *Journal of Molecular Biology*.

[14] Liu B., Xu J., Lan X. (2014). iDNA-Prot|dis: identifying dna-binding proteins by incorporating amino acid distance-pairs and reduced alphabet profile into the general pseudo amino acid composition. *PLoS ONE*.

[15] Fang Y., Guo Y., Feng Y., Li M. (2008). Predicting DNA-binding proteins: approached from Chou's pseudo amino acid composition and other specific sequence features. *Amino Acids*.

[16] Kumar K. K., Pugalenthi G., Suganthan P. N. (2009). DNA-prot: identification of DNA binding proteins from protein sequence information using random forest. *Journal of Biomolecular Structure and Dynamics*.

[17] Lou W., Wang X., Chen F., Chen Y., Jiang B., Zhang H. (2014). Sequence based prediction of DNA-binding proteins based on hybrid feature selection using random forest and Gaussian naïve Bayes. *PLoS ONE*.

[18] Liu B., Xu J., Fan S., Xu R., Zhou J., Wang X. (2015). PseDNA-Pro: DNA-binding protein identification by combining Chou's PseAAC and physicochemical distance transformation. *Molecular Informatics*.

[19] Dong Q., Wang S., Wang K., Liu X., Liu B. Identification of DNA-binding proteins by auto-cross covariance transformation.

[20] Wei L., Tang J., Zou Q. (2017). Local-DPP: an improved DNA-binding protein prediction method by exploring local evolutionary information. *Information Sciences*.

[21] Xu R., Zhou J., Wang H., He Y., Wang X., Liu B. (2015). Identifying DNA-binding proteins by combining support vector machine and PSSM distance transformation. *BMC Systems Biology*.

[22] Im J., Tuvshinjargal N., Park B., Lee W., Huang D.-S., Han K. (2015). PNImodeler: web server for inferring protein-binding nucleotides from sequence data. *BMC Genomics*.

[23] Paz I., Kligun E., Bengad B., Mandel-Gutfreund Y. (2016). BindUP: a web server for non-homology-based prediction of DNA and RNA binding proteins. *Nucleic Acids Research*.

[24] Shanahan H. P., Garcia M. A., Jones S., Thornton J. M. (2004). Identifying DNA-binding proteins using structural motifs and the electrostatic potential. *Nucleic Acids Research*.

[25] Nimrod G., Schushan M., Szilágyi A., Leslie C., Ben-Tal N. (2010). iDBPs: a web server for the identification of DNA binding proteins. *Bioinformatics*.

[26] Xu R., Zhou J., Liu B. (2015). Identification of DNA-binding proteins by incorporating evolutionary information into pseudo amino acid composition via the top-n-gram approach. *Journal of Biomolecular Structure and Dynamics*.

[27] Zhao X.-W., Li X.-T., Ma Z.-Q., Yin M.-H. (2012). Identify DNA-binding proteins with optimal Chou's amino acid composition. *Protein and Peptide Letters*.

[28] Lyons J., Dehzangi A., Heffernan R. (2015). Advancing the accuracy of protein fold recognition by utilizing profiles from hidden Markov models. *IEEE Transactions on NanoBioscience*.

[29] Chou K.-C. (2011). Some remarks on protein attribute prediction and pseudo amino acid composition. *Journal of Theoretical Biology*.

[30] Remmert M., Biegert A., Hauser A., Söding J. (2012). HHblits: lightning-fast iterative protein sequence searching by HMM-HMM alignment. *Nature Methods*.

[31] Liu B., Fang L., Liu F. (2015). Identification of real microRNA precursors with a pseudo structure status composition approach. *PLoS ONE*.

[32] Berman H. M., Westbrook J., Feng Z. (2006). The protein data bank. *International Tables for Crystallography Volume F: Crystallography of biological macromolecules*.

[33] Dondoshansky I., Wolf Y. (2002). *Blastclust (NCBI Software Development Toolkit)*.

[34] Kuchibhatla D. B., Sherman W. A., Chung B. Y. W. (2014). Powerful sequence similarity search methods and in-depth manual analyses can identify remote homologs in many apparently ‘orphan’ viral proteins. *Journal of Virology*.

[35] UniProt Consortium (2017). Uniprot: the universal protein knowledgebase. *Nucleic Acids Research*.

[36] Taguchi Y.-H., Gromiha M. M. (2007). Application of amino acid occurrence for discriminating different folding types of globular proteins. *BMC Bioinformatics*.

[37] Sharma A., Lyons J., Dehzangi A., Paliwal K. K. (2013). A feature extraction technique using bi-gram probabilities of position specific scoring matrix for protein fold recognition. *Journal of Theoretical Biology*.

[38] Altschul S. F., Madden T. L., Schäffer A. A. (1997). Gapped BLAST and PSI-BLAST: a new generation of protein database search programs. *Nucleic Acids Research*.

[39] Sharma R., Dehzangi A., Lyons J., Paliwal K., Tsunoda T., Sharma A. (2015). Predict gram-positive and gram-negative subcellular localization via incorporating evolutionary information and physicochemical features into chou's general PseAAC. *IEEE Transactions on NanoBioscience*.

[40] Sharma A., Paliwal K. K., Dehzangi A., Lyons J., Imoto S., Miyano S. (2013). A strategy to select suitable physicochemical attributes of amino acids for protein fold recognition. *BMC Bioinformatics*.

[41] Dehzangi A., Sohrabi S., Heffernan R. (2015). Gram-positive and gram-negative subcellular localization using rotation forest and physicochemical-based features. *BMC Bioinformatics*.

[42] Dehzangi A., Sharma A., Lyons J., Paliwal K. K., Sattar A. (2014). A mixture of physicochemical and evolutionarybased feature extraction approaches for protein fold recognition. *International Journal of Data Mining and Bioinformatics*.

[43] Dehzangi A., Paliwal K., Lyons J., Sharma A., Sattar A. Enhancing protein fold prediction accuracy using evolutionary and structural features.

[44] Powers D. M. Evaluation: from precision, recall and f-measure to roc, informedness, markedness and correlation.

[45] Efron B., Gong G. (1983). A leisurely look at the bootstrap, the jackknife, and cross-validation. *The American Statistician*.

[46] Pedregosa F., Varoquaux G., Gramfort A. (2011). Scikit-learn: machine learning in Python. *Journal of Machine Learning Research*.

[47] Thompson J. D., Gibson T. J., Higgins D. G. (2002). Multiple sequence alignment using ClustalW and ClustalX. *Current Protocols in Bioinformatics*.

[48] Peng H., Long F., Ding C. (2005). Feature selection based on mutual information: criteria of max-dependency, max-relevance, and min-redundancy. *IEEE Transactions on Pattern Analysis and Machine Intelligence*.

[49] Zou Q., Zeng J., Cao L., Ji R. (2016). A novel features ranking metric with application to scalable visual and bioinformatics data classification. *Neurocomputing*.

